# Loneliness associates strongly with anxiety and depression during the COVID pandemic, especially in men and younger adults

**DOI:** 10.1038/s41598-022-13049-9

**Published:** 2022-06-09

**Authors:** Olivier D. Steen, Anil P. S. Ori, Klaas J. Wardenaar, Hanna M. van Loo

**Affiliations:** 1 Department of Psychiatry, University Medical Center Groningen, University of Groningen, Hanzeplein 1, PO Box 30.001, 9700 RB Groningen, The Netherlands; 2 Department of Genetics, University Medical Center Groningen, University of Groningen, Groningen, The Netherlands

**Keywords:** Anxiety, Depression, Human behaviour

## Abstract

Loneliness is associated with major depressive disorder (MDD), and likely also with generalized anxiety disorder (GAD). It is unclear if these associations are moderated by age, sex, or genetic susceptibility for MDD. We included 75,279 individuals from the Lifelines COVID-19 study, a longitudinal study of a Dutch population-based cohort. Participants completed up to sixteen digital questionnaires between March 2020 and January 2021, yielding a total of 616,129 observations. Loneliness was assessed with the Three-Item Loneliness Scale, and MDD and GAD with the Mini-International Neuropsychiatric Interview. We used generalized estimating equations to investigate the association between loneliness and MDD and GAD, and whether this association varied across time, age, sex and MDD polygenic risk. Loneliness was strongly associated with all MDD and GAD outcomes. Individuals with the highest loneliness scores were around 14 times more likely to have MDD, and 11 times more likely to have GAD, compared to individuals who reported the least loneliness. The association between loneliness and MDD symptoms was stronger in men, younger individuals, and increased across time. While MDD polygenic risk predicted MDD and GAD outcomes, we did not find an interaction effect with loneliness. Our study, which is the largest to date, confirms that loneliness is an important risk factor for MDD, GAD, depressive and anxiety symptoms, especially in men and younger individuals. Future studies should investigate the mechanisms of these associations and explore loneliness-based interventions to prevent and treat MDD and GAD.

## Introduction

Major depressive disorder (MDD) and generalized anxiety disorder (GAD) are two of the most common mental illnesses worldwide, with substantial morbidity and mortality^1,2^. Gaining a better understanding of the mechanisms that underlie their development is important to reduce the burden of these illnesses. The identification of modifiable risk factors is of particular interest because they provide targets for intervention.

The current COVID-19 pandemic has highlighted the importance of loneliness as a modifiable risk factor for MD^3^. Loneliness is the subjective negative experience of deficient social relationships and is distinct from objective social isolation^4^. Research on loneliness and depression indicates a bidirectional relationship between both constructs, with greater experienced loneliness predicting future depressive symptoms, and vice versa^5^. Furthermore, loneliness influences the course of MDD, impeding the chances of recovery^6^. Recent findings furthermore suggest an association between loneliness and GAD as well, but fewer studies have been performed^7,8^.

However, it is not clear which groups are most vulnerable to the effect of loneliness on depression and anxiety. Different factors may moderate this association, including sex, age and genetic susceptibility. Findings regarding age are mixed. Some studies suggest an increased vulnerability in younger individuals, but few studies included sufficient numbers of adolescents and children to reliably demonstrate this^9,10^. Whether sex moderates the effect of loneliness is also unclear. One study found the effect of loneliness on depression to be more pronounced in men^5^, while others did not observe a moderating effect^9^. Thus, it remains inconclusive whether age or sex moderate the association between loneliness and depression and anxiety outcomes.

Furthermore, the association between loneliness and depressive and anxiety outcomes may be influenced by genetic factors. Loneliness, MDD, and GAD have moderate heritability, which means that genetic factors contribute to their aetiology^11–13^. Genome-wide association studies (GWAS) have identified multiple overlapping regions in the genome which are associated with both experiencing loneliness^14^ and developing MDD^15,16^. This genetic overlap suggests that loneliness and depression could have a shared genetic aetiology, or that individuals with a higher genetic risk for MDD could be more sensitive to developing depression after exposure to loneliness.

The COVID-19 pandemic offers a unique situation to study the relationship of loneliness with depression and anxiety. The COVID-19 pandemic, and subsequent lockdowns, have had a profound impact on social relationships and increased the prevalence of loneliness^3,17,18^. This is akin to a natural experiment: a large share of the population is subjected to sustained loneliness, and not only subjects with a psychiatric history or other specific groups. Some studies have investigated the impact of loneliness on anxiety and depression amidst the COVID pandemic, and report an association between loneliness and anxiety and depression^19^, with young women most at risk^20^, but their findings are challenging to interpret due to the use of unrepresentative and smaller samples. The present study makes use of the Lifelines Cohort Study, which represents a large multi-generational population-based study in the North of the Netherlands with repeated and structured assessments of loneliness, MDD and GAD during the COVID-19 pandemic.

Here, we investigate the association of loneliness with depression and anxiety, as well as moderators of this association, in the largest study to date on this relationship.

## Methods

### Subjects

#### Lifelines COVID-19 cohort

Data were derived from the Lifelines COVID-19 study, a longitudinal extension of the Lifelines cohort^21^. Lifelines is a large multidisciplinary prospective population-based cohort that monitors the health and health-related behaviours in a detailed manner of 167,729 persons living in the North of the Netherlands. The cohort consists primarily (98%) of white Dutch native people^22,23^. All subjects provided written informed consent for participation in Lifelines, and the Medical Ethical Committee of the University Medical Center Groningen approved the study protocol.

Starting on March 30th 2020, Lifelines invited all adult participants of whom an email address was known (n = 139,679) to participate in the first COVID-19 digital questionnaire, which assessed somatic and mental health, COVID-19 infection status, and loneliness, among other domains. The first questionnaire was distributed on March 30, 2020. The first six questionnaires (Q1–Q6) were sent out weekly with items assessing participants' experiences in the 7 days prior to filling out the questionnaire. Starting from Q7, questionnaires were sent biweekly or monthly, with items assessing participants’ experiences in the 14 days prior to assessment (Supplementary Table A). A further description of the Lifelines COVID-19 cohort can be found elsewhere^24^.

Data from the first nineteen consecutive assessments (March 30th, 2020, to January 29th, 2021) were available. Questionnaires eleven, twelve and thirteen did not include measures of loneliness, so data from these measurements were not included in our analyses. A total of n = 75,279 individuals participated in at least one of the remaining sixteen questionnaires and are included in this study. In some instances (n = 14,763), participants completed multiple different assessments on the same date. In this case, we included only the questionnaire completed first.

### Measurements

#### Depression and anxiety outcomes

Symptoms of GAD and MDD were assessed with a self-report digital questionnaire based on the Mini-International Neuropsychiatric Interview (MINI)^25^, which assesses all symptoms that are part of the DSM-IV diagnostic criteria. Symptoms that are part of both MDD and GAD (being easily fatigued, difficulty concentrating and sleep disturbance) were assessed once in every questionnaire to avoid repetition.

The presence of current MDD and GAD were established according to the diagnostic criteria of the DSM-IV. For every questionnaire, we also calculated a sum score of MDD (range 0–9) and GAD (range 0–7) criterion symptoms, resulting in two outcomes (symptoms and diagnoses) for both MDD and GAD.

#### Loneliness

The experience of loneliness was assessed through the previously validated three-item UCLA scale^26^. This scale consists of three items (‘How often do you feel that you lack companionship?’, ‘How often do you feel left out?’ and ‘How often do you feel isolated from others?’), with a three-point (0–2) Likert scale. We calculated a loneliness sum score ranging from 0 to 6, with 6 being the maximum score.

#### Demographic variables

Age was defined as subjects’ age when completing the first questionnaire. The sex variable refers to biological sex assigned at birth, which was determined by linking the Lifelines data to information stored in personal records of the municipalities in the Netherlands.

#### Polygenic risk score

We calculated MDD polygenic risk scores (PRS) in 19,128 subjects who had genotype data available. MDD PRS was constructed as a weighted sum of risk alleles for MDD with weights defined as single nucleotide polymorphism (SNP) effect sizes derived from the meta-analysis of the Psychiatric Genomics Consortium (PGC) and UKBiobank GWASs^15^. The Lifelines sample was not included in the base GWAS, but was used as a target sample to calculate PRS for each participant. Details of PRS calculation are discussed in Supplementary Methods.

### Missing data

While all subjects were invited to participate in all assessments, few completed all 16 assessments. Out of 75,279 subjects, 11,528 (15.3%) completed one assessment, 54,409 (72.3%) completed > 1 and < 16 assessments, and 9342 (12.4%) completed all assessments. As expected, there were thus a large number of non-responses for different time points.

Some data were missing due to design choices of the questionnaire (e.g., suicidal ideation was not assessed in every instance). As the questionnaire was web-based, some data were missing due to technical glitches (e.g., a failing internet connection on the participants’ end). Further details on missing data are provided in the Supplementary Methods, while rates of missing data are reported in Supplementary Table B.

As missing data were limited, we implemented multivariate imputation by chained equation (MICE) to impute missing data in one dataset^27^. For imputation, all available data, including data on loneliness, lifetime history of MDD and GAD and accompanying age of onset and number of experienced episodes, stressful life events, and the NEO personality inventory neuroticism score that were collected in previous Lifelines assessments, were used as predictors in MICE. Items were only imputed if a participant filled out other items on that specific questionnaire. Questionnaires with a non-response (i.e. complete missing data) were not imputed. Detailed imputation parameters are presented in Supplementary Methods.

### Statistical methods

#### Model specification

We used generalized estimating equations (GEE) to investigate the association between loneliness and the four depression and anxiety outcomes. GEE is a technique for estimating parameters of a generalized linear model with correlated measurements, yielding population-averaged coefficients. Because our data included repeated measurements within subjects, we used an exchangeable working correlation structure to account for the dependence of measurements within individuals. A further description of GEE can be found elsewhere^28^.

We performed GEE Poisson regression for the sum scores of MDD and GAD symptoms as both of these outcomes were count-variables. We used GEE logistic regression for the dichotomous outcomes of MDD and GAD. The fitted models included a linear and squared effect of time to account for seasonality. Furthermore, we included loneliness, age and sex as main effects. We added an interaction term between loneliness and time to investigate a possible change in the impact of loneliness on MDD and GAD outcomes across the study duration. We furthermore added interaction terms between loneliness and age, and loneliness and sex (main model) to identify possible moderating effects of age and/or sex. For the model including PRS in participants with genetic data available, we also added a main effect for MDD polygenic risk to the model, as well as an interaction term of PRS with loneliness. Furthermore, for the model including PRS, we included ten principal components as model terms to account for population structure confounding.

Multiple testing correction was implemented by Bonferroni correction. We conducted ten hypothesis tests across four outcomes, yielding a total of 40 hypothesis tests and a Bonferroni-corrected alpha of 0.00125. We performed GEE using the *‘geepack’* R package version 1.3.2^29^, and all analyses were performed in R version 4.0.3^30^.

### Sensitivity analyses

#### Attrition bias

Individuals experiencing much loneliness, or who suffer from mental illnesses, may more likely be lost to attrition in longitudinal studies^31^. To determine if results were sensitive to attrition, we conducted a sensitivity analysis with individuals who participated at least three times across three intervals covering the entire study duration. As assessment dates were variable, we constructed these intervals in such a way that the number of assessments was equal across intervals. The first time interval covered the period March 30, 2020 until 29 April 2020. The second interval covered the interval of 30 April 2020 until 10 July 2020. The third interval covered 10 July 2020 until 31 January 2021. 42,001 (55.8%) participants had completed at least one assessment in each of the three intervals and were included in this sensitivity analysis. Participants in this subsample completed a median of 13 [IQR 10–15] responses.

#### Family structure

Lifelines is a multi-generational sample and includes family members, which means that our longitudinal data were not only nested within individuals, but also within families. GEE cannot appropriately account for correlated measurements across multiple levels of clustering^28^. Therefore, to test whether our results were influenced by pedigree structures, we conducted a sensitivity analysis in a subsample consisting of one randomly selected subject for every family. As there were 42,089 pedigrees part of the present study, this analysis included 42,089 (55.9%) subjects.

For both sensitivity analyses, we estimated models in the full sample, and in the sample with PRS data available. Because only a limited number of participants had PRS data available, subsamples in the sensitivity analyses are comparatively small (n = 10,955 and n = 7036), and possibly underpowered to detect interaction effects. The latter was therefore only used to test the robustness of a possible interaction term between loneliness and PRS.

### Ethics approval and consent to participate

All subjects provided written informed consent for participation in Lifelines, and the Medical Ethical Committee of the University Medical Center Groningen approved the study protocol. All methods were carried out in accordance with relevant guidelines and regulations.

## Results

### Characteristics of the study population

**Figure 1:**
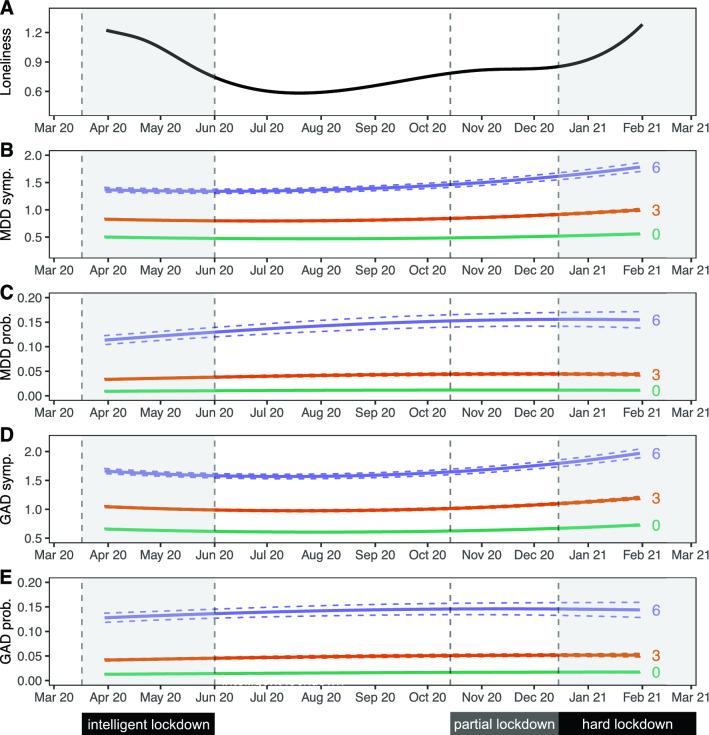
Trajectories of loneliness and MDD and GAD outcomes over time. Shaded areas represent periods during which lockdown measures were enforced in the Netherlands. The first 'intelligent' lockdown consisted of restrictions such as social distancing and bans on public gatherings. During the partial lockdown, some restrictions were reintroduced. The 'hard' lockdown introduced more restrictions and a curfew. (**A**) Marginal GEE effect plot of a GEE model of loneliness scores predicted by a polynomial spline time term. The 95% confidence interval of model-predicted values is not visible because the interval is very small. (**B**–**E**) Marginal GEE effect plot of predicted counts of MDD symptoms (**B**), odds of MDD diagnoses (**C**), counts of GAD symptoms (**D**) and odds of GAD diagnoses (**E**) across different levels (0/green; lowest, 3/orange; moderate, 6/purple; highest) of loneliness scores. Predicted values represent symptoms counts and diagnosis probabilities, and are based on main model fits (Tables 2 and 3). Dashed lines represent a 95% confidence interval of model-predicted values.

The 75,279 individuals included in this study completed a total of 616,129 assessments across 16 time points. Out of these individuals, 19,128 (25.4%) had genotype data available. The median number of collected questionnaires per individual was 8 (IQR 3–14) (Table 1). The mean age at first assessment was 53.7 (standard deviation 12.9). The majority of participants in the sample were female (60.8%), which is similar to the full Lifelines cohort^23^. MDD was present in 5442 (7.2%) participants and GAD in 6733 (8.9%) participants in the 1/2 weeks preceding at least one questionnaire assessment. Loneliness scores were highest during the start and end of data collection, corresponding to periods of nationwide lockdown as a result of government action in response to the COVID-19 pandemic (Fig. 1A).

**Table 1:** Characteristics of study population.

	Full sample	Subsample with genotype data available
n	75,279	19,128
No. questionnaires (median [IQR])	8 [3, 14]	8 [3, 14]
Age (mean ± SD)	53.7 (12.95)	52.4 (13.86)
**Sex (%)**		
Male	29,472 (39.2)	7220 (37.7)
Female	45,807 (60.8)	11,908 (62.3)
Lifetime MDD (%)	17,527 (23.3)	4309 (22.5)
Lifetime GAD (%)	6890 (9.2)	1691 (8.8)
MDD reported in at least one questionnaire (%)	5442 (7.2)	1269 (6.6)
GAD reported in at least one questionnaire (%)	6733 (8.9)	1615 (8.4)

### Loneliness is associated with MDD and GAD symptoms and diagnoses

We observed an association between loneliness and MDD and GAD. We found that a one-point increase in loneliness was associated with a 55.9% increased odds of MDD, and a 48.9% increased odds of GAD. This means that the group scoring highest on loneliness had a 14.4 times increased odds of MDD and a 10.9 times increased odds of GAD compared to those with the lowest loneliness scores (Tables 2 and 3, Fig. 1C,E). Sensitivity analyses showed that these effects were robust for attrition and family structures (Supplementary Tables D1-3 and E1-3).

**Table 2:** Table of GEE models for outcome of MDD symptoms and diagnoses.

	MDD symptoms		MDD diagnoses	
	Main model	Model including PRS^a^	Main model	Model including PRS^a^
Terms	IRR [95% CI]	IRR [95% CI]	OR [95% CI]	OR [95% CI]
Time	0.992 [0.991, 0.994]*	0.990 [0.987, 0.993]*	1.016 [1.009, 1.022]*	1.020 [1.007, 1.034]
Time^2^	1.000 [1.000, 1.000]*	1.000 [1.000, 1.000]*	1.000 [1.000, 1.000]*	1.000 [0.999, 1.000]
Loneliness	1.275 [1.251, 1.300]*	1.271 [1.225, 1.320]*	1.559 [1.469, 1.654]*	1.539 [1.365, 1.734]*
Lone × time	1.001 [1.000, 1.001]*	1.001 [1.000, 1.001]	1.001 [1.000, 1.001]	1.001 [1.000, 1.002]
Lone × age	0.999 [0.999, 0.999]*	0.999 [0.999, 1.000]	1.000 [0.999, 1.002]	1.002 [0.999, 1.004]
Lone × sex	0.971 [0.961, 0.981]*	0.964 [0.945, 0.985]*	0.967 [0.936, 0.998]	0.920 [0.859, 0.986]
Lone × MDD PRS		0.994 [0.985, 1.002]		0.978 [0.950, 1.006]
Age	0.982 [0.981, 0.983]*	0.983 [0.981, 0.985]*	0.967 [0.964, 0.971]*	0.965 [0.958, 0.971]*
Female sex	1.397 [1.357, 1.437]*	1.367 [1.288, 1.450]*	1.228 [1.112, 1.356]*	1.311 [1.055, 1.631]
MDD PRS		1.116 [1.087, 1.145]*		1.265 [1.151, 1.391]*
Constant	0.980 [0.923, 1.040]	0.894 [0.799, 1.001]	0.050 [0.041, 0.061]*	0.043 [0.030, 0.063]*
N	75,279	19,128	75,279	19,128
CIC	16.222	32.889	15.8	32.793

Displayed are incidence rate ratios (IRR) and odds ratios (OR) with 95% confidence intervals for the different terms in the GEE models. Asterisks indicate statistical significance at Bonferroni-corrected α = 0.00125. All models include a linear and squared time term, a main loneliness effect and a loneliness-time interaction. Furthermore, terms for age and female sex are included both as main effect and a loneliness interaction term. The models in genotyped samples also include an MDD polygenic risk score both as main effect and as a loneliness interaction. N refers to the number of individuals included in the dataset. The correlated information criterion (CIC) is a measure of fit for GEE models.

^a^In sample with genotype data available. Models include the first 10 principle components of the genotype data as covariates as well (not shown).

**Table 3:** Table of GEE models for outcome of GAD symptoms and diagnoses.

	GAD symptoms		GAD diagnoses	
	Main model	Model including PRS^a^	Main model	Model including PRS^a^
Terms	IRR [95% CI]	IRR [95% CI]	OR [95% CI]	OR [95% CI]
Time	0.991 [0.989, 0.992]*	0.988 [0.985, 0.991]*	1.014 [1.008, 1.020]*	1.016 [1.003, 1.029]
Time^2^	1.000 [1.000, 1.000]*	1.000 [1.000, 1.000]*	1.000 [1.000, 1.000]	1.000 [0.999, 1.000]
Loneliness	1.199 [1.180, 1.218]*	1.184 [1.148, 1.221]*	1.489 [1.412, 1.570]*	1.384 [1.244, 1.541]*
Lone × time	1.000 [1.000, 1.000]	1.000 [1.000, 1.001]	0.999 [0.999, 1.000]	1.000 [0.998, 1.001]
Lone × age	1.000 [1.000, 1.000]	1.000 [1.000, 1.001]	1.001 [1.000, 1.002]	1.002 [1.000, 1.004]
Lone × sex	0.979 [0.971, 0.988]*	0.980 [0.963, 0.998]	0.969 [0.942, 0.998]	0.975 [0.916, 1.038]
Lone × MDD PRS		0.995 [0.988, 1.002]		0.989 [0.963, 1.015]
Age	0.979 [0.978, 0.979]*	0.979 [0.977, 0.980]*	0.968 [0.965, 0.971]*	0.965 [0.960, 0.970]*
Female sex	1.401 [1.365, 1.439]*	1.360 [1.289, 1.436]*	1.331 [1.222, 1.448]*	1.208 [1.008, 1.449]
MDD PRS		1.105 [1.080, 1.131]*		1.200 [1.109, 1.298]*
Constant	1.603 [1.522, 1.688]*	1.515 [1.373, 1.671]*	0.062 [0.052, 0.073]*	0.064 [0.047, 0.088]*
N	75,279	19,128	75,279	19,128
CIC	14.948	29.368	14.629	29.811

Displayed are incidence rate ratios (IRR) and odds ratios (OR) with 95% confidence intervals for the different terms in the GEE models. Asterisks indicate statistical significance at Bonferroni-corrected α = 0.00125. All models include a linear and squared time term, a main loneliness effect and a loneliness-time interaction. Furthermore, terms for age and female sex are included both as main effect and a loneliness interaction term. The models in genotyped samples also include an MDD polygenic risk score both as main effect and as a loneliness interaction. N refers to the number of individuals included in the dataset. The correlated information criterion (CIC) is a measure of fit for GEE models.

^a^In sample with genotype data available. Models include the first 10 principle components of the genotype data as covariates as well (not shown).

We found a similar association between loneliness and symptom severity of both MDD and GAD, which was robust across both sensitivity analyses (Tables 2 and 3; Fig. 1B,D; Supplementary Tables D1-3 and E1-3). A one-point increase in loneliness was associated with a 27.5% increase in the number of MDD symptoms, and a 19.9% increase in the number of GAD symptoms. This means that the group scoring highest on loneliness had on average 4.3 times as many MDD symptoms, and 3.0 times as many GAD symptoms, compared to the group with the lowest loneliness score.

We observed a significant interaction between loneliness and time, meaning that the magnitude of the association between loneliness and MDD symptoms increased slightly across time (Tables 2 and 3, Fig. 1B,D). A 1 week increment in time was associated with a 0.06% increase in effect size, which corresponds to a 2.4% stronger association between loneliness and MDD symptoms after 10 months (roughly the duration covered by the repeated questionnaires). This interaction effect remained significant in both sensitivity analyses. Taken together, these analyses demonstrate a strong association between loneliness and MDD and GAD outcomes.

### Moderators of the association between loneliness and depression

**Figure 2:**
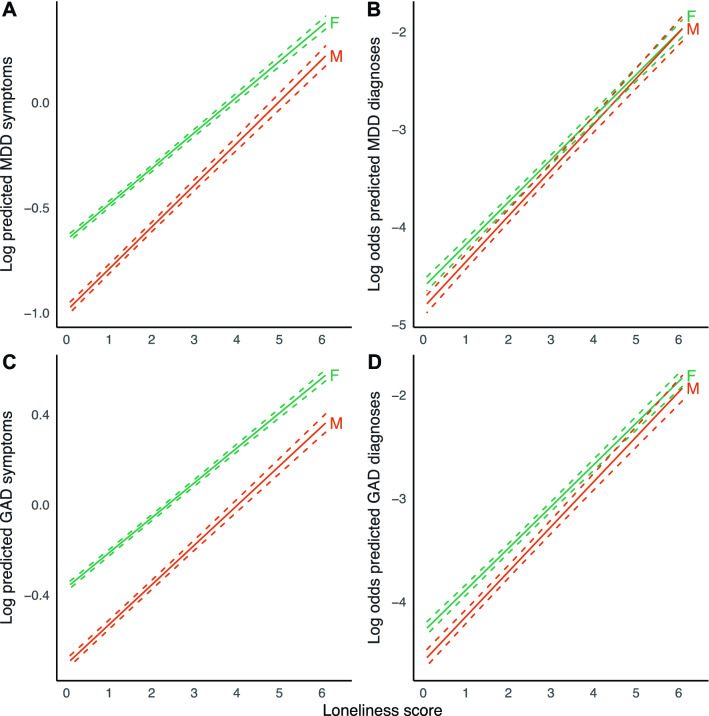
Marginal effect plots of associations between loneliness and MDD and GAD outcomes across sex. Displayed are log predicted counts of MDD symptoms (**A**) and log odds of MDD diagnosis (**B**), as well as log predicted counts of GAD symptoms (**C**) and log odds of GAD (**D**), across different loneliness scores, for men (M) and women (F) (plotted estimates are derived from Tables 2 and 3, main models). Dashed lines represent 95% confidence intervals of model-predicted values.

We found a significant interaction effect of loneliness and sex, indicating that the association between loneliness and MDD symptoms was stronger in men than in women (Table 2, Fig. 2). This effect was consistent across both sensitivity analyses. We observed a similar result for GAD symptoms, but this was not robust in the sensitivity analysis for attrition. We performed a sex-stratified analysis for MDD symptoms to estimate effect sizes for men and women separately (Supplementary Table C5). Results were in agreement with the main analysis, with a point increase in loneliness score being associated with a 32.1% increase in MDD symptoms in men, compared to 22.5% in women. These analyses suggest that the association between loneliness and symptoms of MDD is stronger in men.

**Figure 3:**
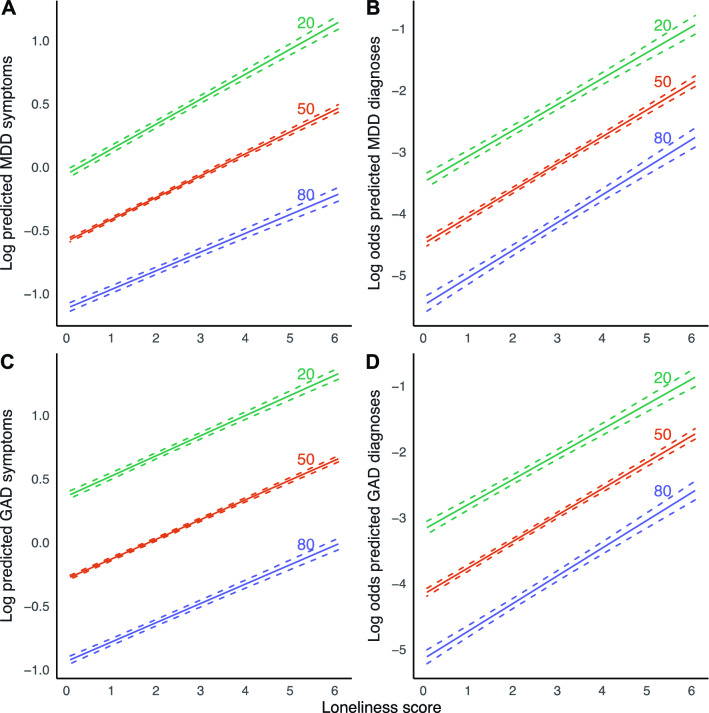
Marginal effect plots of associations between loneliness and MDD and GAD outcomes across age. Displayed are log predicted counts of MDD symptoms (**A**) and log odds of MDD diagnosis (**B**), as well as log predicted counts of GAD symptoms (**C**) and log odds of GAD (**D**), across different loneliness scores, across three levels of age in years (plotted estimates are derived from Tables 2 and 3, main models). Dashed lines represent 95% confidence intervals of model-predicted values.

We found a significant interaction effect between loneliness and age for MDD symptoms (Table 2, Fig. 3), indicating that the magnitude of the association between loneliness and MDD symptoms increased slightly across age. This finding was robust across both sensitivity analyses. A 1 year increment in age was associated with a 0.08% reduction in effect size, which corresponds to a 4.7% weaker association between loneliness and MDD symptoms with a 60-year age difference. A point increase in loneliness is then associated with an increase in the number of MDD symptoms of 25.5% in 20-year-olds, compared to 19.6% in 80-year-olds. We observed no moderating effect of age for the other outcomes.

We found a significant main effect of PRS on symptoms of both MDD and GAD, as well MDD and GAD diagnoses, with a one standard deviation increase in PRS Z-score being associated with 26.5% increased odds of MDD, and 20.0% increased odds of GAD. We however did not observe a modifying effect of PRS on the association between loneliness and the four outcomes in any of the models.

## Discussion

### Summary of main findings

This study investigated the association between loneliness and (symptoms of) MDD and GAD, and whether this association differs across age, sex and MDD PRS. We found significant associations between loneliness and MDD, GAD, MDD symptoms and GAD symptoms. On average, individuals who experienced the most loneliness were about 14 times more likely to report MDD, and around 11 times more likely to report GAD compared to individuals experiencing the least loneliness. For MDD symptoms, the strength of this association increased across time, becoming ~ 2.4% stronger between April 2020 and February 2021.

The association between loneliness and symptoms of MDD was significantly stronger in men than in women. The association of loneliness and MDD symptoms was also stronger in younger individuals. MDD polygenic risk did not modify the association between loneliness and the different outcomes.

### Interpretation of findings and comparison with previous studies

We found a robust association between loneliness and MDD symptom severity. We found a similar association between loneliness and MDD. These findings are in line with multiple previous population-based studies^5,9,32^, which we now replicate in, to our knowledge, the largest study conducted to date. The strength of the association between loneliness and symptoms of MDD, but not MDD diagnoses, increased across the study duration. This could be due to several reasons. It may, for example, suggest that, as the COVID-19 pandemic lingered on, its influence magnified the effect of loneliness on specific depressive symptoms, but that this increase in symptoms did not necessarily cause a participant to develop MDD. Future studies could test this hypothesis, and assess if loneliness associates with only specific depressive symptoms. Alternatively, although the depressive symptom score and MDD are positively associated, they are distinct phenotypic constructs. MDD will likely display less variation across the period of data collection compared to MDD symptoms. Furthermore, the association of the two constructs with loneliness may also be different. Lastly, the present study may be underpowered to detect a significant interaction term for dichotomous outcomes (MDD), compared to continuous outcomes (MDD symptoms). However, we think that our sample of > 75,000 individuals and > 600,000 measurements should be large enough to trace any interaction effect with a relevant effect size.

We also found a robust association between loneliness and symptoms of GAD as well as GAD diagnoses, which is in line with previous studies^7,8^. This could mean that loneliness is a risk factor for GAD, or it could mean that GAD is a risk factor for loneliness or both. We however did not find that this association changed over time, suggesting that the course of the COVID-19 pandemic did not impact the relationship between loneliness and (symptoms of) GAD.

We found that the association between loneliness and depressive symptoms was stronger in men than in women. Results in previous studies were inconsistent. Cacioppo et al. found a stronger association in men in a cross-sectional study of 1939 older adults^5^. However, Lee et al. did not find a moderation effect of sex in a longitudinal study with baseline loneliness as a predictor and depressive symptoms at follow-up as an outcome, in a sample of 4211 older adults^9^. The present study assessed loneliness and outcomes across multiple contemporaneous timepoints. It may be that the moderating effect of sex disappears in time-lagged scenarios—possibly, loneliness has a stronger transient impact in men, without having a stronger effect on sustained depressive symptoms or disorders. Another explanation is that previous studies may not have been sufficiently powered to consistently detect a moderating effect of sex. We stress the importance of conducting such analyses in large population-based samples. Furthermore, differences in study populations, such as demographic, social, and environmental factors could contribute to different study outcomes as well.

We found a modifying effect of younger age on the association between loneliness and MDD symptoms. One previous study conducted in 1006 adults observed a significant interaction effect between loneliness and age on both anxiety and depressive symptoms^10^, while this effect was not found in a sample of 4211 adults above 50 years of age^9^. A lack of young adults in these previous studies may have contributed to these mixed findings. As MDD and GAD often first develop in adolescents or young adults, it is warranted that this age group is included in study cohorts, as is the case for the Lifelines cohort.

The present study did not find a significant moderation effect of MDD PRS on the association between loneliness and anxiety or depression. As far as we know, no comparable studies exist. A previous study by Lee et al. did assess the impact of both loneliness and MDD PRS on the association between loneliness and depression, but only as a confounder, not as an interaction term with loneliness. The authors found that the effect of loneliness persisted also in a model adjusted for PRS^9^. PRS for MDD currently explains only a limited amount of variance (Nagelkerke’s R^2^ 1.5–3.2%) of MDD^15^, thereby capturing only a small proportion of the heritability. As PRS does not capture the complete genetic architecture of MDD, we cannot exclude that genetic factors may impact the relationship between loneliness and MDD and GAD outcomes. Furthermore, while loneliness and depression share genes and heritability, both constructs likely also have phenotype-specific heritability, while the present study only employed genetic susceptibility for MDD. Perhaps, a PRS for loneliness or a PRS calculated from gene variants shared between the two constructs might be a significant moderator. Future work should revisit such analyses as sample sizes of GWASs, continue to increase. Finally, genetic analyses are worthwhile to explore for GAD and loneliness PRS as well, especially now that larger GWASs have been conducted^14,33^.

While loneliness is strongly associated with MDD and GAD outcomes, many individuals experiencing significant loneliness do not develop MDD or GAD. While age and sex explain some of these differences, future research should include other variables as well to further understand how loneliness impacts MDD and GAD. These might include socioeconomic variables, specific environmental variables, or psychological traits related to (tolerance of) loneliness and isolation.

### Strengths and limitations

A strength of the present study is its large sample size and longitudinal design, with repeated measures of loneliness and depression and anxiety outcomes that are measured using multi-item and well-validated instruments.

As our study was conducted amidst the COVID-19 pandemic in the North of the Netherlands, it raises the question of whether our results are generalizable to populations not or no longer affected by the pandemic or to other regions or countries. We think results may be robust, given that the effect sizes of the association between loneliness and MDD and GAD outcomes are comparable to those found in studies conducted before the pandemic and in other populations^5,9^.

Because of the initial weekly and later biweekly/monthly assessments of symptoms, we only assessed MDD and GAD symptoms during the past week or 2 weeks. The DSM requires GAD symptoms to be present for 6 months, which means that our GAD diagnoses likely do not correspond to those of the cases seen in clinical practice. A similar point can be made for MDD diagnoses, which require 14 days of symptoms according to the DSM.

The present study focused on contemporaneous associations but did not investigate temporal relations between loneliness and MDD or GAD. While previous longitudinal studies showed that loneliness predicts (symptoms of) MDD^9^, and vice versa^5^, such studies have not yet been performed for GAD. In addition, studies involving loneliness-targeted interventions can further elucidate the causal relevance of loneliness towards depression and anxiety outcomes, besides evaluating its value as a target for treatment.

As we imputed one single dataset, we have sub-optimally accounted for the uncertainty introduced by our missing data handling approach^34^. However, as we had limited missing data and most data were missing by design (i.e., missing completely at random), we deem it unlikely to have significantly influenced our results.

The present study used a GEE marginal model as opposed to a conditional approach such as generalized linear mixed-effects models. The latter approach also allows the estimation of population-averaged estimates and can adjust for multiple correlation structures (such as for correlation within both individuals and families). This approach however was not feasible as it required excessive computational resources given our large sample size. However, in this study, the estimates from GEE models are likely to be in line with results that would have been derived from conditional models. First, estimates obtained from a GEE model agree closely with those from a conditional model if the right assumptions are being met, as is the case for the present study^35^. Second, our sample size was large, which means that we had sufficient power to trace small effects, even with GEE.

### Implications

The current study replicates that loneliness is strongly associated with MDD and demonstrates a significant association in a large population-based sample for GAD. We furthermore observed that the association between loneliness and MDD symptoms became stronger over time. As COVID-19-related restrictions have led to more loneliness in the population, this might precipitate a subsequent increase in diagnoses, possibly even as government restrictions are being loosened. Our findings warrant extra vigilance in groups such as younger individuals, who experienced more loneliness.

Besides its association with MDD and GAD, loneliness is strongly predictive of a myriad of adverse health outcomes, such as cardiovascular disease and mortality^36^. Currently, few effective interventions are used routinely in clinical practice or the community, while effective interventions do exist^37,38^. Furthermore, there is a stigma surrounding loneliness^39^, and it has not received significant attention in clinical practice or policy. This is unfortunate, as its amelioration could entail a large health gain across somatic and mental domains. If nationwide restrictions on social relationships can lead to more loneliness and a higher prevalence of depression and anxiety in the population, public health policies aimed at nurturing social interactions may achieve the reverse. Some interventions already exist^37,38^, but have not yet been widely employed in clinical or public health settings.

## Conclusions

In the largest study on loneliness and mental health to date, we found that loneliness is strongly associated with MDD, GAD and the symptoms thereof during the COVID-19 pandemic in the Netherlands. The association between loneliness and symptoms of MDD was stronger in men and in younger adults. Finally, we found the association between loneliness and symptoms of MDD to become stronger over time during the COVID-19 pandemic.

## Supplementary Information

Supplementary Information.

## Data Availability

All data is available through the Lifelines Cohort Study. Application for data access can be sent to the Lifelines Research Office: https://www.lifelines.nl/researcher/how-to-apply.
