# Supplementary material for: Loneliness associates strongly with anxiety and depression during the COVID pandemic, especially in men and younger adults
Source: Sci Rep. 2022 Jun 9;12:9517. doi: 10.1038/s41598-022-13049-9 (PMC9178936; doi:10.1038/s41598-022-13049-9)
Supplement: Supplementary file 1 — Supplementary Information. [file 41598_2022_13049_MOESM1_ESM.pdf]

Supplementary Methods & Supplementary Tables

Manuscript title: Loneliness associates strongly with anxiety and depression during the COVID pandemic, especially in men and younger adults

Author list:
Olivier D. Steen BSc
Anil P. S. Ori MSc
Klaas J. Wardenaar PhD
Lifelines Corona Research Initiative
the Lifelines Cohort Study
Hanna M. van Loo MD PhD

Supplementary Methods

Polygenic risk score calculation

To calculate polygenic risk scores, we used weights from the meta-analysis of the Psychiatric Genomics Consortium (PGC) and UKBiobank GWASs [15], for which the GWAS summary statistics were downloaded from the PGC website (https://www.med.unc.edu/pgc/download-results/). The Lifelines sample, which was not included in the base GWAS, was used as a target sample. The genotyping of the Lifelines sample was performed under the umbrella of the UMCG Genetics Lifelines Initiative (UGLI). In total, 38,030 participants were genotyped using the Infinium Global Screening Array® (GSA) MultiEthnic Disease Version 1.0. After stringent sample quality control, genotype data were available for 34,286 participants, of which 19,128 also participated in the present study. Genotypes were imputed on the Sanger Imputation service using the Haplotype Reference Consortium (http://www.haplotype-reference-consortium.org) panel using standard protocol. After imputation, there were 6,183,769 genetic markers. PRS were calculated using PLINK v2.0 [2].We first selected high-quality SNPs by including only markers with less than 5% marker missing rate (--geno 0.05) and that were in Hardy-Weinberg equilibrium (--hwe 1e-6). We further selected SNPs that were also HapMap3 sites with MAF > 1% in Lifelines. SNPs were then clumped (--clump-p1 1, --clump-r2 0.1, --clump-kb 250) to select for a total of 264,071 independent markers. Finally, PRS were calculated using the --score function with a GWAS P-value threshold of 0.05 and subsequently standardized to a mean of zero and unit variance of 1 using the scale() function in R.

Missing data

We define two types of missing data. The first type concerns questionnaire items of MINI-fatigue and MINI-suicidality that were not assessed in all questionnaires. The MINI-fatigue item was not assessed in Q1-Q9 and first included in Q10, because other fatigue items were included in earlier questionnaires. The MINI item on suicidal ideation was assessed once per month to limit the burden on participants. For both these items, we assume data to be missing completely at random because they were based on design choices of the questionnaire. The second type of missing data concerns non-response to specific items on questionnaires that study participants filled out. This can arise due to technical glitches in the web portal or participants accidentally not answering a questionnaire item for example. This type of missing data is generally rare (Supplementary Table B).

MICE imputation parameters

Predictors were determined with the quickpred() function by selecting predictors with data available on at least 25% of participants (minpuc=0.25) and with an absolute correlation threshold of 0.08 or higher (mincor=0.08). Imputation was then performed using the mice() function with the following specified parameters m=1, maxit=1, nnet.MaxNWts=5000. This yielded one imputation batch of complete cases that were used for statistical analyses. Imputation was performed using the mice v3.13 package [41] in R v4.0.3 [30].

Additional references:

40. Chang, C. C. *et al.* Second-generation PLINK: rising to the challenge of larger and richer datasets. *Gigascience* **4**, s13742-015 (2015).
41. Buuren, S. van & Groothuis-Oudshoorn, K. mice: Multivariate imputation by chained equations in R. *J. Stat. Softw.* 1–68 (2010).

Supplementary Tables

The following tables are presented on the following pages:

A. Questionnaire dates *5*

B. Imputation statistics *6*

C. Main analysis

C1-4. GEE effect sizes of stepwise models *7-10*

C5. Sex-stratified analysis *11*

D. Sensitivity analysis for attrition

D1. Characteristics of study population *12*

D2-3. Tables of effect sizes *13-14*

E. Sensitivity analysis for family structure

E1. Characteristics of study population *15*

E2-3. Tables of effect sizes *16-17*

Supplementary Table A: Questionnaire dates

Date refers to the first moment questionnaire invites were sent. Questionnaires were available for completion for 3 weeks. *: Questionnaires 11, 12 & 13 were not included in the present study.

**1**	30/03/2020
**2**	06/04/2020
**3**	13/04/2020
**4**	20/04/2020
**5**	27/04/2020
**6**	04/05/2020
**7**	18/05/2020
**8**	01/06/2020
**9**	15/06/2020
**10**	06/07/2020
**11***	12/07/2020
**12***	10/08/2020
**13***	07/09/2020
**14**	12/10/2020
**15**	02/11/2020
**16**	16/11/2020
**17**	30/11/2020
**18**	14/12/2020
**19**	11/01/2021

Supplementary Table B: Imputation statistics

*: values presented are mean Likert scores (range 1-3) instead of symptom prevalences.

	prevalence pre-imputation (%)	% missing pre-imputation	prevalence post-imputation (%)	% missing post-imputation	% change
**A1 depressed mood**	3	0.99	3	0	-0.07
**A2 anhedonia**	4.46	1.01	4.47	0	-0.19
**A3A weight or appetite change**	6.37	0.93	6.37	0	-0.04
**A3B sleep problems**	19.15	0.95	19.17	0	-0.09
**A3C psychomotor agitation/retardation**	3.26	0.97	3.26	0	0
**A3D fatigue**	8.31	63.97	8.72	0	-4.94
**A3E worthlessness or guilt**	2.19	1	2.19	0	-0.01
**A3F difficulty concentrating**	5.36	1.07	5.37	0	-0.12
**A3G suicidality**	0.74	51.84	0.73	0	2.28
**O1 excessive worry**	3	1.24	3.02	0	-0.75
**O3A restless**	9.52	0.92	9.53	0	-0.04
**O3B feeling tense**	13.93	0.99	13.93	0	-0.02
**O3E irritable**	8.48	1.11	8.48	0	0
**loneliness_1**	1.22*	1.04	1.22*	0	-0.01
**loneliness_2**	1.47*	1.09	1.47*	0	0
**loneliness_3**	1.26*	1.22	1.26*	0	-0.01

Supplementary Table C1: Stepwise GEE models for the outcome of MDD symptoms
Displayed are incidence rate ratios (IRR) with 95% confidence intervals for the different terms in the GEE model. Asterisks indicate statistical significance at Bonferroni-corrected α = 0.00125. Model 1 models linear and squared time and a main loneliness effect, while model 2 includes a loneliness main and loneliness-time interaction effect. Models 3, 4 and 5 respectively add age, female sex and a polygenic risk score to the model, both as a main effect as well as a loneliness-interaction. N refers to the number of individuals included in the model dataset. The correlated information criterion (CIC) is a measure of fit for GEE models. †: In sample with genotype data available. Models include the first 10 principle components of the genotype data as covariates as well (not shown).

	**Model 1**	**Model 2**	**Model 3**	**Model 4**	**Model 5†**
**Terms**	IRR [95% CI]	IRR [95% CI]	IRR [95% CI]	IRR [95% CI]	IRR [95% CI]
**Time**	0.993 [0.991, 0.994]*	0.992 [0.990, 0.993]*	0.992 [0.991, 0.994]*	0.992 [0.990, 0.993]*	0.990 [0.987, 0.993]*
**Time^2**	1.000 [1.000, 1.000]*	1.000 [1.000, 1.000]*	1.000 [1.000, 1.000]*	1.000 [1.000, 1.000]*	1.000 [1.000, 1.000]*
**Loneliness**	1.207 [1.201, 1.212]*	1.197 [1.190, 1.203]*	1.244 [1.223, 1.265]*	1.217 [1.206, 1.228]*	1.196 [1.183, 1.209]*
**Lone x Time**		1.001 [1.000, 1.001]*	1.001 [1.000, 1.001]*	1.001 [1.000, 1.001]*	1.001 [1.000, 1.001]*
**Lone x Age**			0.999 [0.999, 1.000]*		
**Lone x Female Sex**				0.973 [0.963, 0.982]*	
**Lone x MDD PRS**					0.994 [0.986, 1.003]
**Age**			0.981 [0.980, 0.982]*		
**Female sex**				1.470 [1.429, 1.513]*	
**MDD PRS**					1.115 [1.087, 1.145]*
**Constant**	0.474 [0.467, 0.481]*	0.481 [0.473, 0.488]*	1.297 [1.228, 1.369]*	0.375 [0.365, 0.384]*	0.454 [0.440, 0.468]*
**N**	75,279	75,279	75,279	75,279	19,128
**CIC**	7.09	9.46	12.808	12.946	26.383

Supplementary Table C2: Stepwise GEE models for the outcome of MDD diagnoses

Displayed are odds ratios (OR) with 95% confidence intervals for the different terms in the GEE model. Asterisks indicate statistical significance at Bonferroni-corrected α = 0.00125. Model 1 models linear and squared time and a main loneliness effect, while model 2 includes a loneliness main and loneliness-time interaction effect. Models 3, 4 and 5 respectively add age, female sex and a polygenic risk score to the model, both as a main effect as well as a loneliness-interaction. N refers to the number of individuals included in the model dataset. The correlated information criterion (CIC) is a measure of fit for GEE models. †: In sample with genotype data available. Models include the first 10 principle components of the genotype data as covariates as well (not shown).

	**Model 1**	**Model 2**	**Model 3**	**Model 4**	**Model 5†**
**Terms**	OR [95% CI]	OR [95% CI]	OR [95% CI]	OR [95% CI]	OR [95% CI]
**Time**	1.014 [1.009, 1.020]*	1.013 [1.007, 1.019]*	1.016 [1.010, 1.022]*	1.013 [1.007, 1.019]*	1.016 [1.004, 1.029]
**Time^2**	1.000 [1.000, 1.000]	1.000 [1.000, 1.000]	1.000 [1.000, 1.000]*	1.000 [1.000, 1.000]	1.000 [0.999, 1.000]
**Loneliness**	1.569 [1.547, 1.591]*	1.553 [1.526, 1.580]*	1.514 [1.435, 1.598]*	1.591 [1.547, 1.635]*	1.539 [1.482, 1.598]*
**Lone x Time**		1.001 [1.000, 1.001]	1.001 [1.000, 1.001]	1.001 [1.000, 1.001]	1.001 [1.000, 1.003]
**Lone x Age**			1.001 [1.000, 1.002]		
**Lone x Female Sex**				0.962 [0.933, 0.991]	
**Lone x MDD PRS**					0.978 [0.951, 1.007]
**Age**			0.966 [0.963, 0.970]*		
**Female sex**				1.356 [1.230, 1.496]*	
**MDD PRS**					1.261 [1.149, 1.385]*
**Constant**	0.010 [0.009, 0.011]*	0.010 [0.010, 0.011]*	0.059 [0.050, 0.071]*	0.008 [0.008, 0.009]*	0.009 [0.008, 0.010]*
**N**	75,279	75,279	75,279	75,279	19,128
**CIC**	7.098	8.866	12.223	12.524	26.587

Supplementary Table C3: Stepwise GEE models for the outcome of GAD symptoms

Displayed are incidence rate ratios (IRR) with 95% confidence intervals for the different terms in the GEE model. Asterisks indicate statistical significance at Bonferroni-corrected $\alpha = 0.00125$. Model 1 models linear and squared time and a main loneliness effect, while model 2 includes a loneliness main and loneliness-time interaction effect. Models 3, 4 and 5 respectively add age, female sex and a polygenic risk score to the model, both as a main effect as well as a loneliness-interaction. N refers to the number of individuals included in the model dataset. The correlated information criterion (CIC) is a measure of fit for GEE models. †: In sample with genotype data available. Models include the first 10 principle components of the genotype data as covariates as well (not shown).

	**Model 1**	**Model 2**	**Model 3**	**Model 4**	**Model 5†**
**Terms**	IRR [95% CI]	IRR [95% CI]	IRR [95% CI]	IRR [95% CI]	IRR [95% CI]
**Time**	0.990 [0.989, 0.992]*	0.990 [0.989, 0.991]*	0.991 [0.989, 0.992]*	0.990 [0.989, 0.991]*	0.988 [0.985, 0.990]*
**Time^2**	1.000 [1.000, 1.000]*	1.000 [1.000, 1.000]*	1.000 [1.000, 1.000]*	1.000 [1.000, 1.000]*	1.000 [1.000, 1.000]*
**Loneliness**	1.179 [1.174, 1.183]*	1.175 [1.169, 1.180]*	1.179 [1.163, 1.196]*	1.189 [1.180, 1.198]*	1.171 [1.161, 1.181]*
**Lone x Time**		1.000 [1.000, 1.000]	1.000 [1.000, 1.000]	1.000 [1.000, 1.000]	1.000 [1.000, 1.001]
**Lone x Age**			1.000 [1.000, 1.000]		
**Lone x Female Sex**				0.979 [0.971, 0.987]*	
**Lone x MDD PRS**					0.995 [0.988, 1.002]
**Age**			0.977 [0.977, 0.978]*		
**Female sex**				1.490 [1.452, 1.530]*	
**MDD PRS**					1.105 [1.079, 1.131]*
**Constant**	0.642 [0.634, 0.651]*	0.646 [0.638, 0.655]*	2.124 [2.028, 2.224]*	0.499 [0.488, 0.511]*	0.626 [0.609, 0.643]*
**N**	75,279	75,279	75,279	75,279	19,128
**CIC**	6.717	8.762	11.804	11.944	23.668

Supplementary Table C4: Stepwise GEE models for the outcome of GAD diagnoses

Displayed are odds ratios (OR) with 95% confidence intervals for the different terms in the GEE model. Asterisks indicate statistical significance at Bonferroni-corrected α = 0.00125. Model 1 models linear and squared time and a main loneliness effect, while model 2 includes a loneliness main and loneliness-time interaction effect. Models 3, 4 and 5 respectively add age, female sex and a polygenic risk score to the model, both as a main effect as well as a loneliness-interaction. N refers to the number of individuals included in the model dataset. The correlated information criterion (CIC) is a measure of fit for GEE models. †: In sample with genotype data available. Models include the first 10 principle components of the genotype data as covariates as well (not shown).

	**Model 1**	**Model 2**	**Model 3**	**Model 4**	**Model 5†**
**Terms**	OR [95% CI]	OR [95% CI]	OR [95% CI]	OR [95% CI]	OR [95% CI]
**Time**	1.009 [1.004, 1.015]*	1.010 [1.005, 1.016]*	1.014 [1.008, 1.020]*	1.010 [1.005, 1.016]*	1.012 [1.000, 1.024]
**Time^2**	1.000 [1.000, 1.000]	1.000 [1.000, 1.000]	1.000 [1.000, 1.000]	1.000 [1.000, 1.000]	1.000 [1.000, 1.000]
**Loneliness**	1.491 [1.472, 1.510]*	1.504 [1.481, 1.527]*	1.451 [1.385, 1.521]*	1.534 [1.495, 1.573]*	1.492 [1.444, 1.541]*
**Lone x Time**		0.999 [0.999, 1.000]	0.999 [0.999, 1.000]	0.999 [0.999, 1.000]	1.000 [0.999, 1.001]
**Lone x Age**			1.001 [1.000, 1.002]		
**Lone x Female Sex**				0.964 [0.938, 0.992]	
**Lone x MDD PRS**					0.989 [0.964, 1.015]
**Age**			0.967 [0.964, 0.969]*		
**Female sex**				1.467 [1.349, 1.594]*	
**MDD PRS**					1.196 [1.107, 1.293]*
**Constant**	0.014 [0.013, 0.015]*	0.014 [0.013, 0.014]*	0.078 [0.068, 0.091]*	0.011 [0.010, 0.012]*	0.013 [0.011, 0.014]*
**N**	75,279	75,279	75,279	75,279	19,128
**CIC**	6.599	8.328	11.301	11.669	23.851

Supplementary Table C5: Table of GEE models for outcome of MDD symptoms stratified by sex
Displayed are incidence rate ratios (IRR) and odds ratios (OR) with 95% confidence intervals for the different terms in the GEE models. Asterisks indicate statistical significance at Bonferroni-corrected α = 0.00125. All models include a linear and squared time term, a main loneliness effect and a loneliness-time interaction. A term for age is included both as main effect and a loneliness interaction term. The models in genotyped samples also include an MDD polygenic risk score both as main effect and as a loneliness interaction. N refers to the number of individuals included in the dataset. The correlated information criterion (CIC) is a measure of fit for GEE models. †: In sample with genotype data available. Models include the first 10 principle components of the genotype data as covariates as well (not shown).

	**Male**		**Female**	
	Main model	Model including PRS†	Main model	Model including PRS†
**Terms**	IRR [95% CI]	IRR [95% CI]	IRR [95% CI]	IRR [95% CI]
**Time**	0.988 [0.985, 0.991]*	0.984 [0.978, 0.990]*	0.994 [0.992, 0.996]*	0.993 [0.989, 0.996]*
**Time^2**	1.000 [1.000, 1.000]*	1.000 [1.000, 1.001]*	1.000 [1.000, 1.000]*	1.000 [1.000, 1.000]*
**Loneliness**	1.321 [1.278, 1.366]*	1.315 [1.231, 1.404]*	1.225 [1.202, 1.249]*	1.218 [1.174, 1.263]*
**Lone x Time**	1.001 [1.000, 1.001]*	1.001 [1.000, 1.002]	1.000 [1.000, 1.001]*	1.001 [1.000, 1.001]
**Lone x Age**	0.998 [0.998, 0.999]*	0.999 [0.998, 1.000]	0.999 [0.999, 1.000]	0.999 [0.999, 1.000]
**Lone x MDD PRS**		0.990 [0.973, 1.007]		0.995 [0.986, 1.005]
**Age**	0.982 [0.980, 0.983]*	0.979 [0.976, 0.983]*	0.983 [0.982, 0.984]*	0.984 [0.982, 0.987]*
**MDD PRS**		1.188 [1.133, 1.247]*		1.087 [1.054, 1.121]*
**Constant**	1.046 [0.945, 1.157]	1.088 [0.897, 1.319]	1.324 [1.242, 1.412]*	1.121 [1.000, 1.257]
**N**	29,472	7,220	45,807	11,908
**CIC**	13.253	30.37	12.497	27.373

Supplementary Table D1: Characteristics of study population for sensitivity analysis testing the impact of attrition

We conducted a sensitivity analysis with individuals who participated in at least one questionnaire across three weighted tertiles of measurement dates.

		Full sample	Sample with genotype data available
**n**		42,001	10,955
**No. questionnaires (median [IQR])**		13 [10, 15]	13 [10, 15]
**Age (mean ±SD)**		56.91 (12.15)	55.94 (13.20)
**Sex (%)**	Male	16,083 (38.3)	3,991 (36.4)
	Female	25,918 (61.7)	6,964 (63.6)
**Lifetime MDD (%)**		9,123 (21.7)	2,290 (20.9)
**Lifetime GAD (%)**		3,474 (8.3)	885 (8.1)
**MDD reported in at least one questionnaire (%)**		3,448 (8.2)	828 (7.6)
**GAD reported in at least one questionnaire (%)**		4,409 (10.5)	1,096 (10.0)

Supplementary Table D2: Table of GEE models for outcome of MDD symptoms and diagnoses in the sensitivity analysis testing the impact of attrition Displayed are incidence rate ratios (IRR) and odds ratios (OR) with 95% confidence intervals for the different terms in the GEE models. Asterisks indicate statistical significance at Bonferroni-corrected $\alpha = 0.00125$. All models include a linear and squared time term, a main loneliness effect and a loneliness-time interaction. Furthermore, terms for age and female sex are included both as main effect and a loneliness interaction term. The models in genotyped samples also include an MDD polygenic risk score both as main effect and as a loneliness interaction. N refers to the number of individuals included in the dataset. The correlated information criterion (CIC) is a measure of fit for GEE models. †: In sample with genotype data available. Models include the first 10 principle components of the genotype data as covariates as well (not shown).

	**MDD symptoms**		**MDD diagnoses**	
	Main model	Model including PRS†	Main model	Model including PRS†
**Terms**	IRR [95% CI]	IRR [95% CI]	OR [95% CI]	OR [95% CI]
**Time**	0.991 [0.989, 0.993]*	0.990 [0.986, 0.994]*	1.014 [1.006, 1.022]*	1.018 [1.002, 1.034]
**Time^2**	1.000 [1.000, 1.000]*	1.000 [1.000, 1.000]*	1.000 [1.000, 1.000]	1.000 [0.999, 1.000]
**Loneliness**	1.251 [1.218, 1.284]*	1.227 [1.169, 1.289]*	1.480 [1.359, 1.613]*	1.373 [1.171, 1.611]*
**Lone x Time**	1.001 [1.000, 1.001]*	1.001 [1.000, 1.002]*	1.001 [1.000, 1.002]	1.002 [1.000, 1.003]
**Lone x Age**	0.999 [0.999, 1.000]*	1.000 [0.999, 1.000]	1.001 [0.999, 1.002]	1.003 [1.000, 1.006]
**Lone x Sex**	0.978 [0.965, 0.990]*	0.970 [0.945, 0.997]	0.987 [0.946, 1.031]	0.935 [0.851, 1.026]
**Lone x MDD PRS**		1.000 [0.989, 1.010]		1.002 [0.967, 1.038]
**Age**	0.983 [0.981, 0.984]*	0.982 [0.979, 0.984]*	0.967 [0.963, 0.972]*	0.960 [0.952, 0.969]*
**Female sex**	1.430 [1.375, 1.487]*	1.418 [1.309, 1.537]*	1.199 [1.049, 1.370]	1.373 [1.013, 1.860]
**MDD PRS**		1.103 [1.066, 1.141]*		1.181 [1.041, 1.339]
**Constant**	0.861 [0.790, 0.939]*	0.846 [0.718, 0.997]	0.046 [0.035, 0.060]*	0.048 [0.028, 0.084]*
**N**	42,001	10,955	42,001	10,955
**CIC**	17.421	33.905	16.875	33.217

Supplementary Table D3: Table of GEE models for outcome of GAD symptoms and diagnoses in the sensitivity analysis testing the impact of attrition
Displayed are incidence rate ratios (IRR) and odds ratios (OR) with 95% confidence intervals for the different terms in the GEE models. Asterisks indicate statistical significance at Bonferroni-corrected $\alpha = 0.00125$. All models include a linear and squared time term, a main loneliness effect and a loneliness-time interaction. Furthermore, terms for age and female sex are included both as main effect and a loneliness interaction term. The models in genotyped samples also include an MDD polygenic risk score both as main effect and as a loneliness interaction. N refers to the number of individuals included in the dataset. The correlated information criterion (CIC) is a measure of fit for GEE models. †: In sample with genotype data available. Models include the first 10 principle components of the genotype data as covariates as well (not shown).

	**GAD symptoms**		**GAD diagnoses**	
	Main model	Model including PRS†	Main model	Model including PRS†
**Terms**	IRR [95% CI]	IRR [95% CI]	OR [95% CI]	OR [95% CI]
**Time**	0.991 [0.989, 0.992]*	0.989 [0.986, 0.992]*	1.014 [1.007, 1.021]*	1.016 [1.002, 1.032]
**Time^2**	1.000 [1.000, 1.000]*	1.000 [1.000, 1.000]*	1.000 [1.000, 1.000]	1.000 [0.999, 1.000]
**Loneliness**	1.183 [1.158, 1.208]*	1.151 [1.106, 1.198]*	1.438 [1.337, 1.547]*	1.277 [1.112, 1.466]*
**Lone x Time**	1.000 [1.000, 1.001]	1.001 [1.000, 1.001]	1.000 [0.999, 1.000]	1.000 [0.998, 1.002]
**Lone x Age**	1.000 [1.000, 1.000]	1.000 [1.000, 1.001]	1.001 [1.000, 1.002]	1.003 [1.001, 1.005]
**Lone x Sex**	0.985 [0.974, 0.996]	0.990 [0.967, 1.013]	0.987 [0.950, 1.025]	0.998 [0.921, 1.081]
**Lone x MDD PRS**		1.000 [0.991, 1.009]		1.003 [0.971, 1.036]
**Age**	0.978 [0.977, 0.979]*	0.977 [0.975, 0.980]*	0.967 [0.963, 0.970]*	0.963 [0.957, 0.970]*
**Female sex**	1.436 [1.385, 1.488]*	1.383 [1.285, 1.488]*	1.313 [1.177, 1.466]*	1.187 [0.938, 1.501]
**MDD PRS**		1.092 [1.059, 1.126]*		1.139 [1.033, 1.256]
**Constant**	1.469 [1.361, 1.586]*	1.483 [1.288, 1.708]*	0.060 [0.048, 0.075]*	0.068 [0.045, 0.104]*
**N**	42,001	10,955	42,001	10,955
**CIC**	15.965	30.294	15.302	30.515

Supplementary Table E1: Characteristics of study population for sensitivity analysis testing the impact of clustering within families

For every family out of a total of 42,089 families included, we randomly selected one individual.

		Full sample	Sample with genotype data available
**n**		42,089	7,036
**No. questionnaires (median [IQR])**		7 [2, 14]	8 [3, 14]
**Age (mean ±SD)**		53.42 (11.95)	52.63 (12.93)
**Sex (%)**	Male	15,938 (37.9)	2,544 (36.2)
	Female	26,151 (62.1)	4,492 (63.8)
**Lifetime MDD (%)**		10,424 (24.8)	1,677 (23.8)
**Lifetime GAD (%)**		4,158 (9.9)	662 (9.4)
**MDD reported in at least one questionnaire (%)**		3,322 (7.9)	508 (7.2)
**GAD reported in at least one questionnaire (%)**		4,100 (9.7)	639 (9.1)

Supplementary Table E2: Table of GEE models for outcome of MDD symptoms and diagnoses in the sensitivity analysis testing the impact of clustering within families.

Displayed are incidence rate ratios (IRR) and odds ratios (OR) with 95% confidence intervals for the different terms in the GEE models. Asterisks indicate statistical significance at Bonferroni-corrected $\alpha = 0.00125$. All models include a linear and squared time term, a main loneliness effect and a loneliness-time interaction. Furthermore, terms for age and female sex are included both as main effect and a loneliness interaction term. The models in genotyped samples also include an MDD polygenic risk score both as main effect and as a loneliness interaction. N refers to the number of individuals included in the dataset. The correlated information criterion (CIC) is a measure of fit for GEE models. †: In sample with genotype data available. Models include the first 10 principle components of the genotype data as covariates as well (not shown).

	**MDD symptoms**		**MDD diagnoses**	
	Main model	Model including PRS†	Main model	Model including PRS†
**Terms**	IRR [95% CI]	IRR [95% CI]	OR [95% CI]	OR [95% CI]
**Time**	0.992 [0.990, 0.994]*	0.990 [0.985, 0.995]*	1.017 [1.009, 1.025]*	1.026 [1.006, 1.046]
**Time^2**	1.000 [1.000, 1.000]*	1.000 [1.000, 1.000]*	1.000 [1.000, 1.000]	1.000 [0.999, 1.000]
**Loneliness**	1.284 [1.250, 1.318]*	1.200 [1.125, 1.279]*	1.556 [1.435, 1.688]*	1.331 [1.088, 1.629]
**Lone x Time**	1.001 [1.000, 1.001]*	1.001 [1.000, 1.002]	1.001 [1.000, 1.002]	1.001 [0.999, 1.003]
**Lone x Age**	0.999 [0.999, 0.999]*	1.000 [0.999, 1.001]	1.000 [0.999, 1.002]	1.003 [1.000, 1.006]
**Lone x Sex**	0.969 [0.957, 0.982]*	0.986 [0.952, 1.022]	0.969 [0.930, 1.010]	0.982 [0.873, 1.104]
**Lone x MDD PRS**		0.993 [0.978, 1.007]		0.997 [0.952, 1.044]
**Age**	0.984 [0.983, 0.985]*	0.983 [0.979, 0.986]*	0.971 [0.966, 0.975]*	0.966 [0.955, 0.976]*
**Female sex**	1.400 [1.348, 1.454]*	1.309 [1.186, 1.445]*	1.244 [1.094, 1.415]*	1.201 [0.841, 1.715]
**MDD PRS**		1.113 [1.066, 1.162]*		1.257 [1.082, 1.461]
**Constant**	0.973 [0.895, 1.057]	1.014 [0.837, 1.227]	0.049 [0.037, 0.064]*	0.050 [0.027, 0.094]*
**N**	42,089	7,036	42,089	7,036
**CIC**	16.302	32.894	15.973	31.856

Supplementary Table E3: Table of GEE models for outcome of GAD symptoms and diagnoses in the sensitivity analysis testing the impact of clustering within families.

Displayed are incidence rate ratios (IRR) and odds ratios (OR) with 95% confidence intervals for the different terms in the GEE models. Asterisks indicate statistical significance at Bonferroni-corrected α = 0.00125. All models include a linear and squared time term, a main loneliness effect and a loneliness-time interaction. Furthermore, terms for age and female sex are included both as main effect and a loneliness interaction term. The models in genotyped samples also include an MDD polygenic risk score both as main effect and as a loneliness interaction. N refers to the number of individuals included in the dataset. The correlated information criterion (CIC) is a measure of fit for GEE models. †: In sample with genotype data available. Models include the first 10 principle components of the genotype data as covariates as well (not shown).

	**GAD symptoms**		**GAD diagnoses**	
	Main model	Model including PRS†	Main model	Model including PRS†
**Terms**	IRR [95% CI]	IRR [95% CI]	OR [95% CI]	OR [95% CI]
**Time**	0.991 [0.989, 0.993]*	0.988 [0.984, 0.992]*	1.014 [1.007, 1.022]*	1.011 [0.992, 1.032]
**Time^2**	1.000 [1.000, 1.000]*	1.000 [1.000, 1.000]*	1.000 [1.000, 1.000]	1.000 [0.999, 1.000]
**Loneliness**	1.211 [1.184, 1.238]*	1.142 [1.084, 1.203]*	1.488 [1.383, 1.601]*	1.291 [1.078, 1.547]
**Lone x Time**	1.000 [1.000, 1.000]	1.000 [1.000, 1.001]	0.999 [0.998, 1.000]	0.999 [0.997, 1.002]
**Lone x Age**	1.000 [0.999, 1.000]	1.000 [0.999, 1.001]	1.001 [0.999, 1.002]	1.002 [0.999, 1.005]
**Lone x Sex**	0.978 [0.967, 0.989]*	1.010 [0.980, 1.040]	0.968 [0.932, 1.004]	1.058 [0.948, 1.179]
**Lone x MDD PRS**		0.999 [0.987, 1.011]		1.029 [0.983, 1.078]
**Age**	0.979 [0.978, 0.981]*	0.978 [0.976, 0.981]*	0.968 [0.964, 0.972]*	0.970 [0.961, 0.979]*
**Female sex**	1.388 [1.341, 1.437]*	1.254 [1.148, 1.369]*	1.310 [1.174, 1.462]*	0.967 [0.724, 1.292]
**MDD PRS**		1.095 [1.053, 1.138]*		1.106 [0.969, 1.263]
**Constant**	1.649 [1.533, 1.775]*	1.750 [1.481, 2.067]*	0.069 [0.055, 0.086]*	0.071 [0.042, 0.120]*
**N**	42,089	7,036	42,089	7,036
**CIC**	15.033	29.089	14.698	30.64
